# Price, quality, and market dynamics of malaria rapid diagnostic tests: analysis of Global Fund 2009–2018 data

**DOI:** 10.1186/s12936-021-04008-2

**Published:** 2022-01-12

**Authors:** Rachel Wittenauer, Spike Nowak, Nick Luter

**Affiliations:** 1 University of Washington School of Public Health, 1959 NE Pacific St, Seattle, WA 98195 USA; 2 PATH, 2201 Westlake Ave, Seattle, WA 98121 USA

**Keywords:** Malaria rapid diagnostic test (RDT), Quality, Unit price, RDT market, Panel detection score, WHO product testing program, Malaria RDT procurement, Global Fund

## Abstract

**Background:**

Rapid diagnostic tests (RDTs) for malaria are a vital part of global malaria control. Over the past decade, RDT prices have declined, and quality has improved. However, the relationship between price and product quality and their larger implications on the market have yet to be characterized. This analysis used purchase data from the Global Fund together with product quality data from the World Health Organization (WHO) and Foundation for Innovative New Diagnostics (FIND) Malaria RDT Product Testing Programme to understand three unanswered questions: (1) Has the market share by quality of RDTs in the Global Fund’s procurement orders changed over time? (2) What is the relationship between unit price and RDT quality? (3) Has the market for RDTs financed by the Global Fund become more concentrated over time?

**Methods:**

Data from 10,075 procurement transactions in the Global Fund’s database, which includes year, product, volume, and price, was merged with product quality data from all eight rounds of the WHO-FIND programme, which evaluated 227 unique RDT products. To describe trends in market share by quality level of RDT, descriptive statistics were used to analyse trends in market share from 2009 to 2018. A generalized linear regression model was then applied to characterize the relationship between price and panel detection score (PDS), adjusting for order volume, year purchased, product type, and manufacturer. Third, a Herfindahl–Hirschman Index (HHI) score was calculated to characterize the degree of market concentration.

**Results:**

Lower-quality RDTs have lost market share between 2009 and 2018, as have the highest-quality RDTs. No statistically significant relationship between price per test and PDS was found when adjusting for order volume, product type, and year of purchase. The HHI was 3,570, indicating a highly concentrated market.

**Conclusions:**

Advancements in RDT affordability, quality, and access over the past decade risk stagnation if health of the RDT market as a whole is neglected. These results suggest that from 2009 to 2018, this market was highly concentrated and that quality was not a distinguishing feature between RDTs. This information adds to previous reports noting concerns about the long-term sustainability of this market. Further research is needed to understand the causes and implications of these trends.

**Supplementary Information:**

The online version contains supplementary material available at 10.1186/s12936-021-04008-2.

## Background

Globally, in 2019 there were an estimated 229 million cases of malaria, including 384,000 deaths [1]. While substantial gains have been made against malaria, especially in the past 20 years, more progress will be required to reach global elimination goals. A critical element to successful malaria case management is prompt, accurate diagnosis of malaria. Prompt diagnosis and treatment is not only vital to patient health, it can reduce costs on the health care system [2], including a major reduction in the presumptive use of artemisinin-based combination therapy [3]. Improving the ability to diagnose malaria quickly and accurately remains a crucial component of the global effort to reduce the burden of malaria on patients and health systems. Historically, this has been conducted through diagnosis by expert microscopists. However, there are limitations to this strategy related to costs, training, and proximity to patients. Rapid diagnostic tests (RDTs) have many advantages over microscopy, including removing the need for instrumentation and reliable electricity, as well as the reduced amount of training needed for RDT use compared to microscopy [4].

In the early 2000s, rapid point-of-care tests began to change the malaria testing landscape, enabling fast test results with minimal training. Availability of rapid tests prompted the World Health Organization (WHO) to change its policy recommendations in 2010, from presumptive treatment to a “test and treat” policy, where patients are only given antimalarial drugs following a positive parasitological diagnosis [5, 6]. The widespread use of RDTs is vital to this policy, and the magnitude of their use has increased rapidly in the past decade due in part to this change in policy [7]. In 2013, diagnosis with RDTs overtook microscopy as the most common tool for diagnosing malaria in sub-Saharan Africa, and by 2018, WHO estimated 412 million RDTs were sold globally, 259 million of which were distributed by national malaria control programmes [8, 9]. Prior to the disruptions to the market caused by COVID-19, estimates from Unitaid projected demand for RDTs would increase 26.3% between 2018 and 2021 based on continued expansion of their use in both the private and public sectors [10]. Despite this expansion of RDT purchasing, large gaps in malaria diagnostics use at the point of care persist. Between 2015 and 2018 in sub-Saharan Africa, the median percentage of febrile children who received a malaria diagnostic test (whether RDT or microscopy) was 66% in the public sector and 40% in the private sector [8], meaning a substantial proportion of febrile children were still not being tested for malaria.

## Malaria RDT procurement and manufacturing

According to the 2020 *World Malaria Report*, US$3 billion was invested in malaria control globally in 2019, US$1.2 billion (39%) of which was disbursed through the Global Fund [1]. Nearly half of the Global Fund’s 2017 disbursements were spent on health product procurement, including RDTs [11]. In 2019, 348 million RDTs were sold by manufacturers [1]. The Global Fund estimates that procurements it finances account for around 65% of the RDT market volume, or in dollar terms approximately US$100 million annually in RDT purchases [12]. The Global Fund Pooled Procurement Mechanism (PPM) and the US President’s Malaria Initiative are the largest institutional buyers, and 20% of the market is made up of RDT purchases made by designated country procurement agents using Global Fund grant dollars [13]. Decisions on which RDTs to purchase with Global Fund financing are either the responsibility of individual ministries of health via country procurement agents or guided by the Global Fund’s PPM, which makes pooled purchases for multiple countries [11, 14]. Whether purchases are directed by health ministries or the PPM, procured RDTs must meet WHO quality standards. One of the mechanisms supporting transparency about affordability of health products such as RDTs is the Global Fund’s Price and Quality Reporting (PQR) platform. A stipulation of a country receiving grants from the Global Fund is that all grant-funded purchases of key pharmaceutical and health products, including malaria RDTs, must be reported to the PQR database [15]. This extensive publicly available database thus provides an “an indicative picture of the range of prices paid by reporting grant recipients” [15].

The WHO supports RDT procurement and quality assurance in multiple ways. In collaboration with the Foundation for Innovative New Diagnostics (FIND) and the US Centers for Disease Control and Prevention, the WHO has historically provided thorough product quality testing of RDTs through its Malaria RDT Product Evaluation Programme. Since its inception in 2008, the programme has evaluated 332 products across the eight rounds of testing, from which all data are published and available publicly [7, 16–22]. The programme was discontinued in 2019, and quality evaluations of RDTs were absorbed by the WHO Prequalification programme. The Prequalification programme will continue to conduct rigorous quality testing and make data on individual RDT performance available, however will no longer publish composite tables comparing RDT quality across products [13, 23]. The results of these evaluations principally provide data on each RDT product’s panel detection scores as a measure of quality (see definition in Methods section) for both *Plasmodium falciparum* and *Plasmodium vivax* detection, as well as false positive rates, thermal stability, and ease of use, among other RDT characteristics. Compliance of RDT procurement with WHO minimum quality standards has been improving since the publication of the first round of product evaluation results, with 26.8% of products meeting all WHO minimum quality standards in 2011 to 79.4% of products by the final evaluation round in 2018 [24]. This shift can be partially attributed to increased awareness of the product testing programme from both the purchaser and the manufacturer side [25].

Despite the vital importance of RDTs to the global control of malaria, several publications in the past decade have highlighted concerns about sustainability in the malaria RDT market, for reasons related to price, quality, market concentration, and trends over time [10, 13, 26]. As one example, malaria RDT manufacturing has recently been dominated by two companies that have made 85% or more of the RDTs since 2013 [13]. The onset of the COVID-19 pandemic in 2020 proved a major stressor on the manufacture of malaria RDTs by these two companies, as SARS-CoV-2 diagnostics took priority. This market shock has sparked continued conversation about long-term market sustainability for diagnostics of global health importance [27, 28].

## Analysis objective

In this paper, data from both Global Fund procurement orders at the transaction level and the WHO-FIND product testing programme at the product level were used to understand the relationship between price and quality of malaria RDTs. Specifically, this analysis aimed investigate three unanswered questions:

1. Has the market share by quality of RDTs changed over time?
2. Is there substantial differentiation in quality given a change in price? That is, what is the relationship between unit price and RDT quality?
3. Has the Global Fund procurement market become more or less concentrated over time?

By examining these relationships using real-world procurement data and independent product quality evaluations, this analysis will provide insight into the relationship between price and quality and the potential dynamics behind concentration in the RDT market. The relationship between price and quality in the global RDT market may also be of interest to RDT manufacturers as they consider designing and pricing products for large-scale purchase by the public sector. The level of concentration in the Global Fund’s procurement orders may also be of interest to large purchasers and funders that want to encourage innovation or ensure sufficiently diversified supply in order to avoid risks that could destabilize the market, such as a large manufacturer exiting the market.

Behind these three aims are three motivating economic concepts that can shed light on the dynamics of the RDT market. The first concept is known as “information asymmetry”: sellers have better information on the quality of the products they are selling than purchasers [29]. Information asymmetry is common for medicines and diagnostics, and trusted arbiters of quality like national drug regulatory bodies and the WHO Prequalification programme have been established to address this issue. In the absence of these trusted sources of information, economic theory predicts that buyers, knowing they are unable to accurately judge the quality of goods, will lower their willingness to pay and poorer-quality products will drive higher-quality products out of the market, potentially to the point where there is no market at all [29]. Through acting as a trusted arbiter of RDT quality information, the WHO-FIND product testing programme should help to overcome the pitfalls of information asymmetry and enable a healthy market for high-quality RDTs.

The second concept is “vertical product differentiation,” which describes markets where products are primarily distinguished from others by variations in quality [30]. In vertically differentiated markets, manufacturers compete primarily on quality and price. In the short run, there may be a wide range of prices corresponding to a wide range in product quality. However, in the long run, manufacturers most able to invest in quality improvements and reach economies of scale will gain market share leading to increased market concentration and reduced variability in product quality and prices [30]. Theoretical predictions of vertically differentiated markets resulting in higher levels of market concentration have been shown empirically in the pharmaceutical industry and others [31–33]. Analyses of the RDT market have revealed the trend toward higher quality and affordability, but they have also raised risks associated with higher levels of market concentration by large manufacturers [14, 13, 26].

The third concept is a measure of market concentration: the Herfindahl–Hirschman Index (HHI). The HHI is commonly used by market regulatory agencies to evaluate the level of competition in a market. For example, the US Department of Justice (US DOJ) uses it to scrutinize potential mergers to ensure they do not lead to an over-concentrated and under-competitive market. The DOJ considers an HHI score of less than 1,500 an unconcentrated market, between 1,500 and 2,500 a moderately concentrated market, and greater than 2,500 a highly concentrated market [34].

Past analyses have demonstrated that the average quality of available RDTs has increased significantly since 2008 [24, 25] and that average unit prices for malaria RDTs have declined in the past decade [13, 35]. However, price and quality have not been examined together across different RDT products, meaning a comparison of purchase prices at the RDT product-specific level (e.g., for products detecting the same species) remains a gap in understanding of the malaria RDT market. The market concentration of RDT manufacturers in the Global Fund’s procurement orders has also not been measured with the HHI.

## Methods

### Data sources and measures

To investigate these three research aims, malaria RDT data from two sources was merged and analysed: (1) the Global Fund PQR public database, and (2) the WHO-FIND product testing evaluations. The Global Fund PQR data contain country-reported transactions from Global Fund–supported programmes, including manufacturer, country, date of purchase, order volume, and price of the order. All prices were reported in US dollars (USD) at the time of order, and this analysis adjusts all dollars to 2018 dollars by using World Bank gross domestic product deflator data for the United States as recommended by the *WHO Guide to Standardization of Economic Evaluations of Immunization Programmes* [36, 37]. The WHO-FIND product evaluations were conducted in eight rounds between 2008 and 2018, and they include data on panel detection score, false positive rates, heat stability, and usability of the product. 

In this investigation, the primary measures of interest were (1) panel detection score (PDS) at 200 parasites/$\upmu$ l for *P. falciparum* and/or *P. vivax* depending on the RDT test line and (2) unit purchase price per RDT. PDS is a surrogate for sensitivity and the main measure of performance used in the WHO-FIND product testing programme evaluation. PDS provides a good indication of adequate analytical sensitivity based on the recommendations made by the product testing programme documentation that “this level is well below the mean parasite density found in many populations in areas with endemic malaria and is considered close to the threshold that must be detected in order to reliably identify clinical malaria in many settings” [7]. Additionally, it is a combined measure of positivity rate and inter-test and inter-lot consistency, rather than a measure of clinical sensitivity, which can vary depending on parasite prevalence in the population of use [7]. Heat stability or false positive rate were not adjusted for because “generally, products with high performance in detecting parasites have low false-positive rates [and] good thermal stability” [7]. For these reasons, PDS was chosen as the measure for RDT quality. For RDT products that include more than one test line (to detect *P. vivax* or other malarias), data from the results of both test lines were used.

Unit price per RDT is drawn from the PQR data, measured in 2018 USD, and calculated as inflation-adjusted order price divided by the volume of RDTs procured. Price per test was adjusted for inflation using World Bank gross domestic product deflator data in USD based on year of purchase [37]. In order to understand price changes in relation to quality of the product at the time, each product was examined based on the quality (PDS) data that was available at the time of its purchase. In several instances, the WHO-FIND product testing evaluations reviewed the same product in different years of testing, and in these instances, the panel detection score that was most recently available at the time of the RDT purchase was used.

The Global Fund PQR data and the WHO-FIND product testing evaluations data were matched on product name and catalog number (Appendix A) after manual review based on the reported product name, catalog numbers (expired and current), manufacturer, and test characteristics. If a product name and information were too ambiguous to determine a match, the product was excluded from the analysis.

**Fig. 1:**
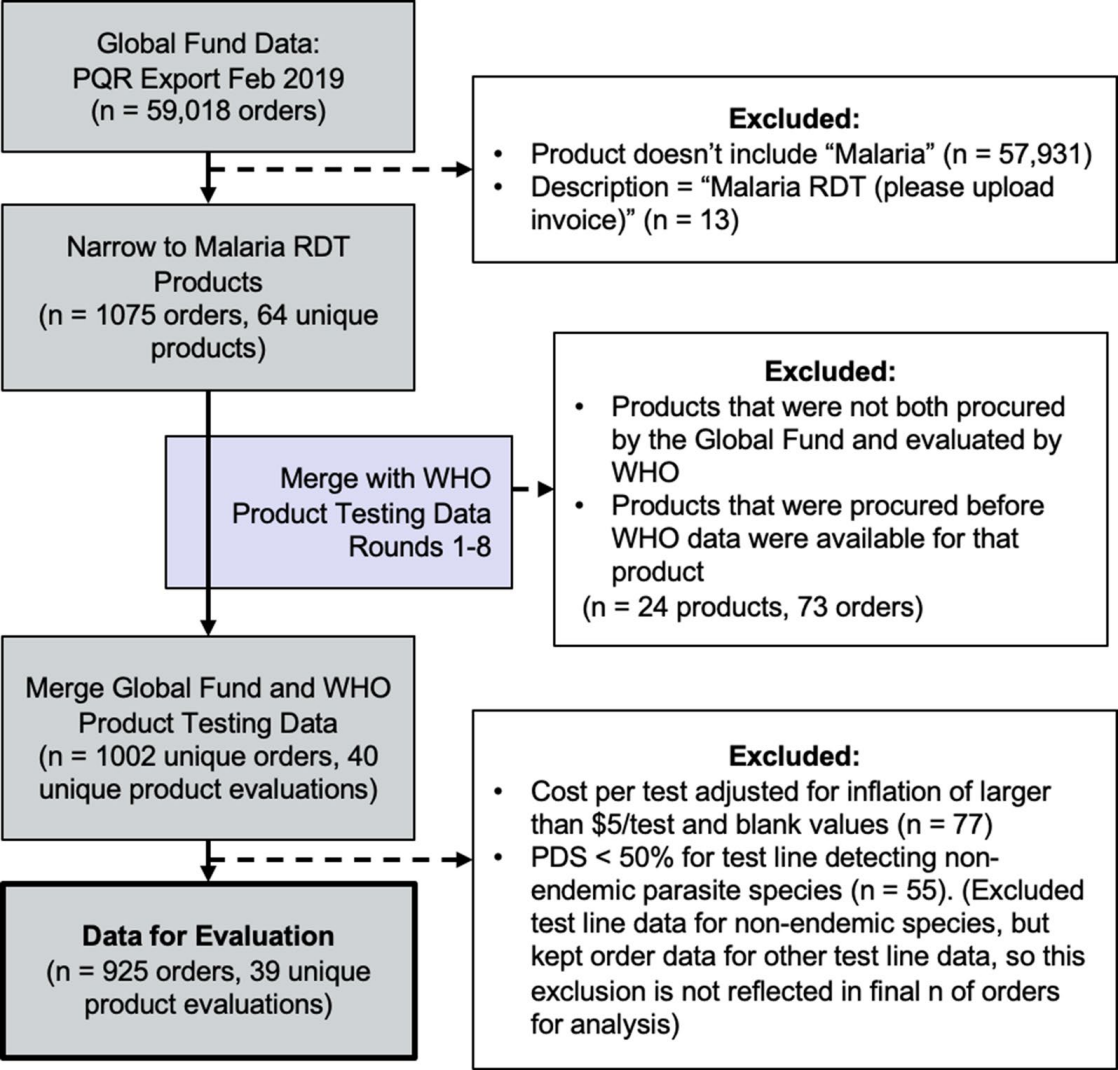
Data included in this analysis are sourced from the Global Fund and the WHO-FIND programme

After joining the PQR and WHO-FIND data, outliers in both price per test and PDS in the resulting dataset were observed. Outliers with price per test greater than ten times the mean price (n = 2) and outliers with a PDS score < 50% for a non-endemic species (n = 55) were removed. Procurement orders that were missing values in the data fields required to calculate price per test (n = 75) were also excluded. Additional detail on outlier identification as well as the complete cleaned dataset are available in Appendices B and D, respectively. This data collection and cleaning process is summarized in Fig. 1. All data sources used in this analysis are available as Additional files and are summarized in Appendix D.

### Sample characteristics

After using the defined exclusion criteria, the dataset for analysis included 925 purchase orders of 39 unique RDT products produced by 11 different manufacturing companies between 2009 and 2018. The total number of individual tests ordered across all six WHO regions was 776,960,035. This set of RDTs is the subset of products that were both evaluated by the WHO-FIND product testing programme and purchased using Global Fund resources, and comprises approximately 37% of the estimated 2.1 billion RDTs procured by the public sector between 2009 and 2018 [13]. The remaining 63% of the RDT market was procured outside of this mechanism. Data on these other segments are largely not publicly available, thus are not included in this analysis. This sample included four types of malaria RDT products: 60.1% of the RDTs purchased were *P. falciparum*-only RDTs, 31.3% were *P. falciparum*/Pan RDTs, 8.5% were *P. falciparum/P. vivax* RDTs, < 0.01% were Pan-only RDTs, and none of the tests were *P. vivax*-only. The majority of RDTs purchased (89.5%) were in the WHO Regional Office for Africa region. The median unit price of an RDT in the total sample was US$0.47, and *P. falciparum*-only tests were the least expensive product type, at a median price per test of US$0.35. The median panel detection score for RDTs detecting *P. falciparum* was 95.0%, and the median PDS for RDTs detecting *P. vivax* was 91.4%. See Tables 1 and 2 for additional sample characteristics.

**Table 1:** Characteristics of the sample by product type and WHO region, 2009 to 2018

Characteristics of the sample by product type and WHO region (2009 to 2018)				
	Number of purchase orders (%)	Total RDT volume (%)	Unique RDT product evaluations^1,2^ (%)	Unique manufacturers^3^ (%)
Total	925 (100%)	776,960,035 (100%)	39 (100%)	11 (100%)
Product type				
P.f	403 (43.6%)	466,737,077 (60.1%)	19 (48.7%)	9 (81.8%)
P.f./Pan	372 (40.2%)	243,217,199 (31.3%)	10 (25.6%)	4 (36.4%)
P.f./P.v	147 (15.9%)	66,414,819 (8.5%)	9 (23.1%)	4 (36.4%)
Pan	3 (< 0.01%)	590,940 (< 0.01%)	1 (2.6%)	1 (9.1%)
WHO region^4^				
AFRO	579 (62.6%)	695,556,122 (89.5%)	29 (74.6%)	7 (63.6%)
SEARO	115 (12.4%)	28,818,510 (3.7%)	18 (46.2%)	6 (54.5%)
EMRO	82 (08.9%)	29,664,394 (3.8%)	15 (38.5%)	3 (27.3%)
WPRO	75 (08.1%)	18,154,870 (2.3%)	9 (23.1%)	3 (27.3%)
AMRO	71 (07.7%)	4,411,119 (< 0.01%)	19 (48.7%)	8 (72.7%)
EURO	3 (< 0.01%)	355,020 (< 0.01%)	3 (7.7%)	1 (9.1%)

^1^ Certain RDTs were evaluated more than once by the WHO product testing programme and PDS scores were updated in the dataset after each round of testing

^2^ Unique RDT product evaluations by region do not total 100% because some RDTs were purchased in multiple regions

^3^ Unique manufacturers by product type and region do not total 100% because some manufacturers produce multiple product types and were purchased in multiple WHO regions

^4^ WHO regions are defined as the Regional Office for Africa (AFRO), the Americas (AMRO), Europe (EURO), the Eastern Mediterranean (EMRO), Southeast Asia (SEARO), and the Western Pacific (WPRO)

**Table 2:** Median unit price and median panel detection score by product type

Median unit price and median panel detection score (PDS) by product type			
	Median unit price of RDT in USD (IQR^1^)	Median P.f. PDS (IQR)	Median P.v. PDS (IQR)
Product type			
P.f (n = 403)	$0.350 ($0.233–$0.591)	96.0 (95.0–98.7)	NA
P.f./Pan (n = 372)	$0.572 ($0.383–$0.836)	92.9 (84.0–96.2)	90.0 (75.0–94.3)
P.f./P.v (n = 147)	$0.509 ($0.427–$0.683)	96.0 (92.0–96.0)	95.0 (94.3–95.0)
Pan (n = 3)	$0.588 ($0.588–$0.591)	84.0 (84.0–84.0)	88.6 (88.6–88.6)
All (n = 925)	$0.473 ($0.323–$0.718)	95.0 (91.0–97.5)	91.4 (75.0–95.0)

^1^ Interquartile range

## Analysis methods

### Aim one: Trends in RDT PDS over time

To investigate the first aim, the trends in RDT PDS over time, the distribution of PDS for each the *P. falciparum* and *P. vivax* panels for each year between 2008 and 2018 were plotted. To understand trends in quality, the RDTs were grouped by their panel detection scores into ranges of PDS: < 75%, 75–79.9%, 80–84.9%, 85–89.9%, 90–95%, and > 95%. Descriptive statistics were used to analyse if market share of RDTs with similar-quality panel detection scores are increasing or losing market share over time.

### Aim two: Relationship between RDT unit price and PDS

To investigate the second aim, the relationship between RDT unit price and PDS, two different linear regression models were fit, as have been used in similar analyses of the malaria RDT market [13, 35]. First (aim 2a), a linear regression model was fitted for each of the *P. falciparum* and *P. vivax* panels, in which changes in price for a given PDS were evaluated, while adjusting for order volume, year of purchase, and product type. In this first model, manufacturer was not adjusted for because the goal was to understand the relationship between price and PDS in the market as a whole, agnostic of manufacturer. If the purchaser were to evaluate a price purely considering quality and cost, variations in manufacturer-specific product lines would not be relevant.

Secondly (aim 2b), manufacturer was included as a covariate in the models to understand relationships between price and quality when controlling for manufacturer. Because of economies of scale, larger manufacturers can charge lower prices than smaller competitors while maintaining profitability, which may cause average prices in the market to be lower and mask relationships between price and quality within each individual manufacturer’s product lines.

In all model interpretations, statistical significance was calculated at the alpha = 0.05 level and robust standard errors were used to correct for heteroscedasticity and non-normality in the data [38].

### Aim three: Market concentration over time

Lastly, to understand market concentration over time, the HHI was calculated, which is expressed in a range from 0 to 1, or with points from 0 to 10,000. It is calculated by summing the squared market share of each firm, and the higher the HHI, the higher the market concentration, as shown in Eq. 1. Market share in Global Fund procurements was defined as percent of the total RDTs procured that were made by a specific manufacturer, divided by the total number of RDTs procured. This calculation was performed for the total set of data as well as for each year of procurement transactions.

Equation 1 The Herfindahl–Hirschman Index

$$HHI=\sum_{i=1}^{N}{s}_{i}^{2}$$

Matching of product names to the PQR database and the WHO-FIND programme results was performed in Microsoft Excel. All other analyses were performed in RStudio version 4.0.2.

## Results

### Aim one: trends in RDT PDS over time

**Fig. 2:**
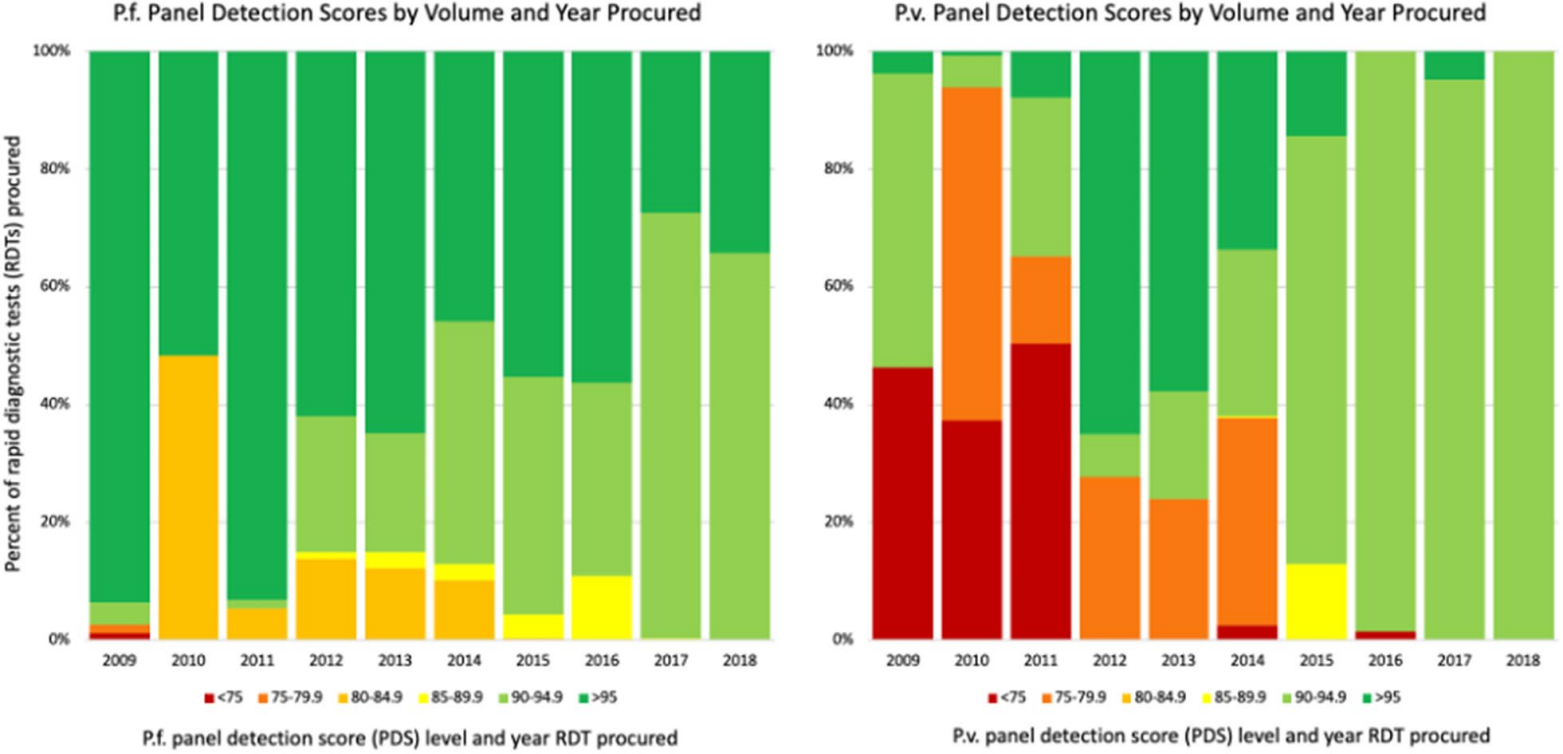
Market share of RDT products by panel detection score (*Plasmodium falciparum* and *Plasmodium vivax*) 2009 to 2018

For the *P. falciparum* panel, in 2009 1% of purchased RDTs were below the current WHO minimum quality standard of 75%, while 94% of all purchased RDTs had a PDS > 95% (the standard was 50% until 2012). In 2013, 0% were below standard, 35% were between 75% and 94.9%, and 65% of RDTs had a PDS of 95% or greater. By 2018, still 0% of purchased RDTs had a PDS below 75%; the majority (66%) had a PDS between 90% and 94.9%, and 34% had a PDS of > 95%. The distribution of *P. vivax* PDS scores among purchased RDTs was similar. Among the *P. vivax–*detecting RDTs, in 2009 46% of RDTs were below the WHO standard of PDS 75% while 50% had scores of 90–94.9% and 4% were 95% or greater. By 2013, 0% of purchased RDTs were below the WHO minimum, 42% were 75–94.9%, and 58% were 95% or greater. By 2018, 100% of purchased RDTs had a PDS of 90–94.9%. These results are summarized in Fig. 2.

By 2018, the majority of the RDT products purchased for both *P. falciparum* and *P. vivax* were of high quality (PDS of 90–94.9%, well above the WHO minimum of 75%). For both *P. falciparum* and *P. vivax parasite* panels, RDTs with panel detection scores below the WHO minimum acceptable threshold of 75% and those barely above (75%–79.9%) have seen their market share in Global Fund procurement orders decrease over time. Along with the lowest-quality tests, tests with the highest PDS scores (PDS > 95%) also appear to be losing Global Fund procurement market share over time—from 65% of the market in 2013 to 34% in 2018 for *P. falciparum*-detecting RDTs, and from 58% in 2013 to 0% in 2018 for *P. vivax*-detecting RDTs.

### Aim two: Relationship between RDT unit price and PDS

**Fig. 3:**
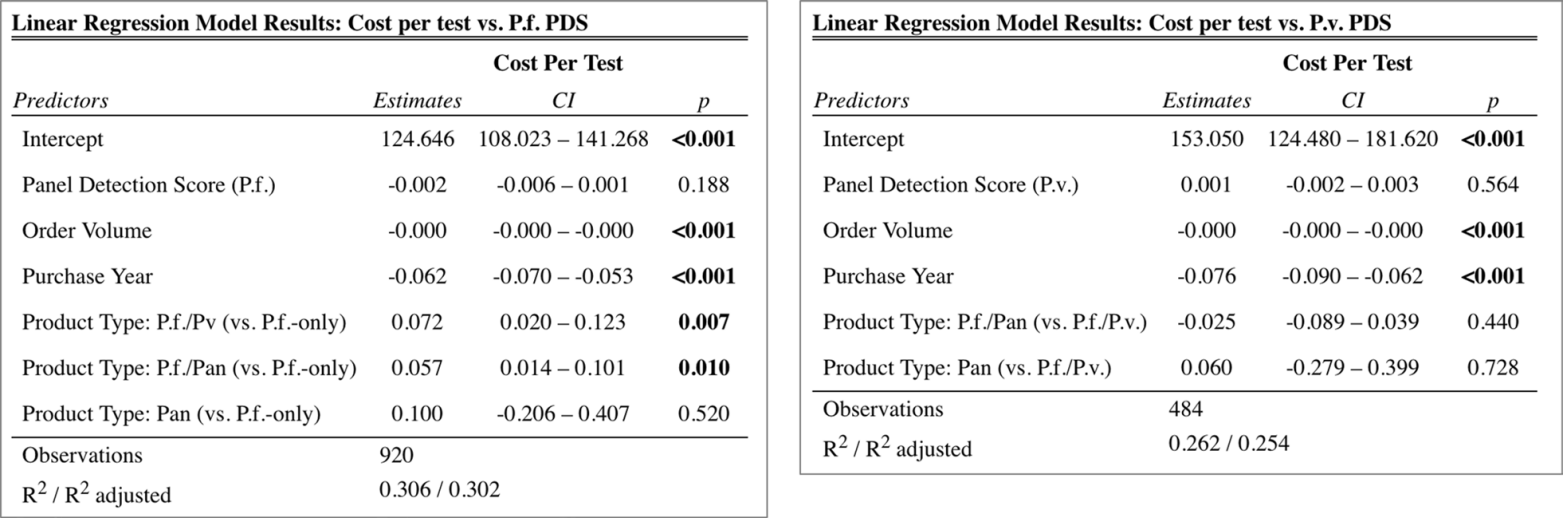
Results of aim 2a regression analyses on cost per test and panel detection score

The adjusted linear regression models showed no significant association between price per test and *P. falciparum* PDS (estimate: – 0.002, p = 0.188) and between price per test and *P. vivax* PDS (estimate: 0.001, p = 0.564). The results are summarized in Fig. 3.

**Fig. 4:**
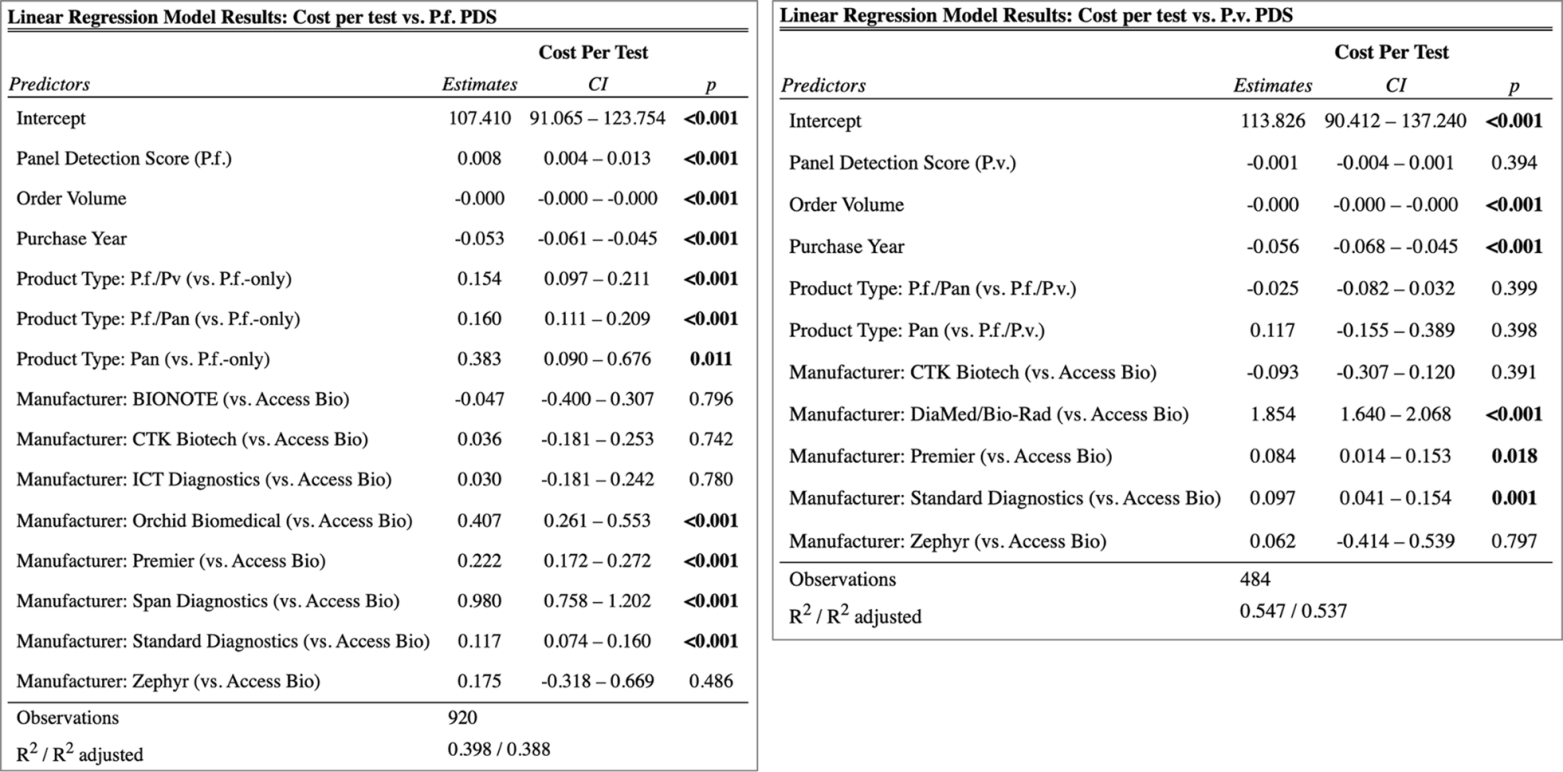
Results of aim 2b regression analysis, with manufacturer included as a covariate

Results from the adjusted linear regression models for aim 2b, including manufacturer as a covariate, found varying relationships as summarized in Fig. 4 below. For RDTs with a *P. falciparum*-detecting test line, there was a statistically significant relationship between price per test and *P. falciparum* PDS (estimate: 0.01, p < 0.001) among RDTs made by the same manufacturer, purchased in the same year and with the same order volume. This estimate corresponds to the trend that for every 1 percentage point increase in PDS, the mean RDT price is 1 cent higher. For RDTs with a *P. vivax*-detecting test line, there was no significant relationship between price per test and PDS (p = 0.394).

Two post-hoc sensitivity analyses for this aim were conducted. In the first, manufacturers with less than 3% of market share in any year were removed, and this analysis found that there was still a significant relationship between price per test and PDS for *P. falciparum*-detecting tests (estimate 0.011, p < 0.001) and still no significant relationship between price per test and PDS for *P. vivax*-detecting tests (p = 0.38). In the second, the “order volume” covariate was replaced with “procurement source” in the model (categorized as PPM, Direct from Manufacturer, or Other) to understand if that mechanism was more relevant than the order volume itself. The results showed there was still no significant association between price per test and PDS for *P. falciparum*-detecting tests (estimate – 0.003, p = 0.056) or *P. vivax*-detecting tests (estimate – 0.0002, p = 0.89). While not statistically significant, the estimates indicate a negative association between price and quality, which is counter to what would be expected if purchasers were willing to pay more for better-quality tests.

### Aim three: Market concentration over time

**Fig. 5:**
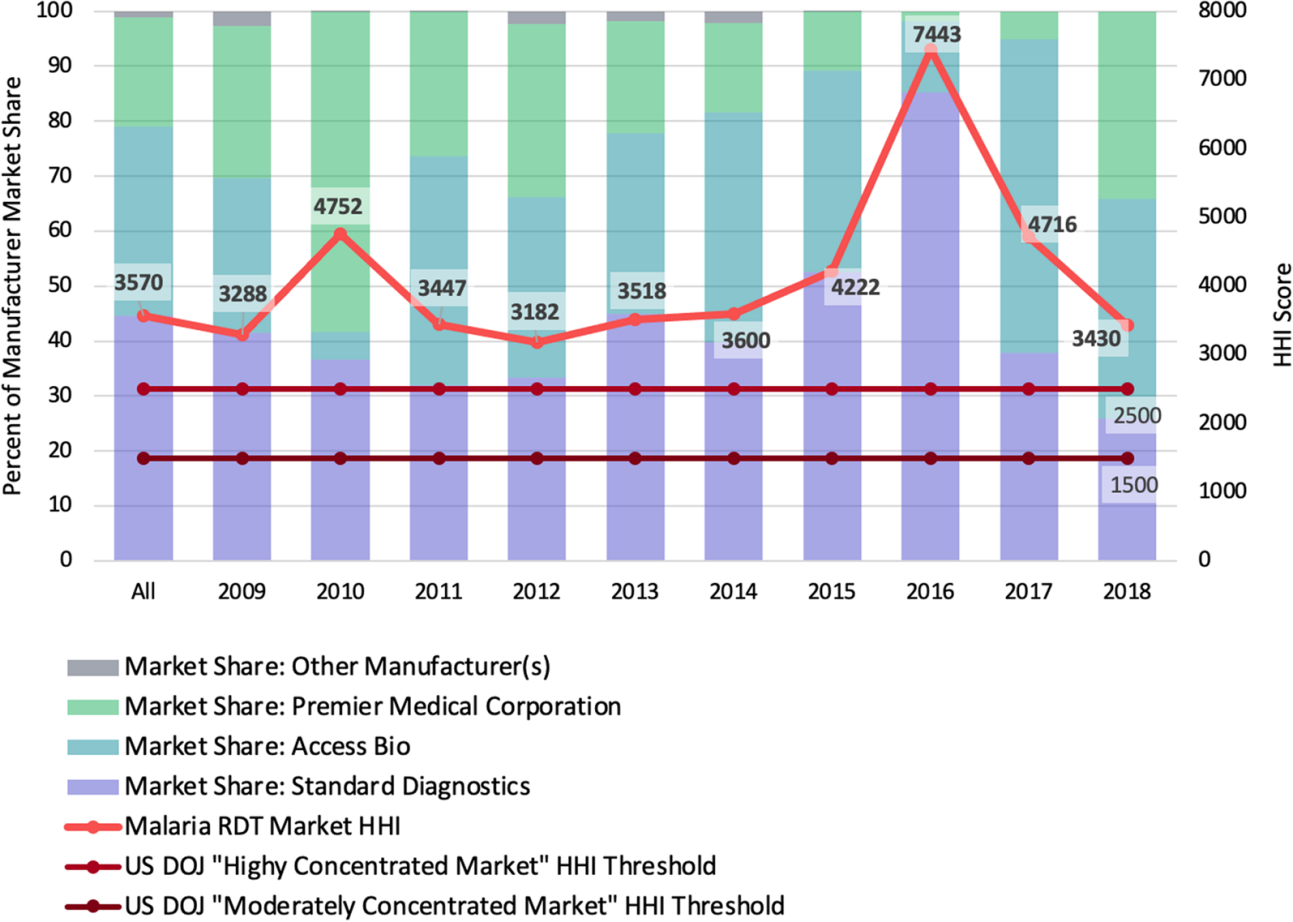
HHI score and market concentration by year

Calculating for the total dataset, the HHI is 3570. The HHI score by year is presented in Fig. 5 below, noting the market share of the top three companies each year. Underlying data including names of manufacturers and their percent market share for each year can be found in Appendix C.

Of note, in each year, the same three companies are the top three manufacturers by market share of procured RDTs. In the last 3 years of analysis, they are the only three manufacturers from which RDTs were procured.

To understand which products remained in the market, the RDT purchase orders placed in 2017 and 2018 were explored post hoc. All were for products from only three manufacturers: Standard Diagnostics (45% of orders), Premier Medical Corporation (37%), and Access Bio (18%). Looking at total RDT volume purchased, however, nearly all (94%) are manufactured by either Access Bio or Standard Diagnostics, as there were a higher number of smaller-volume orders for Premier. This information is summarized in Table 3.

**Table 3:** Characteristics of RDTs purchased in 2017 and 2018

Product Name	Manufacturer	Total Orders	Total RDT Volume	Median Price	Median PDS (P.f. / P.v.)	
First Response® Malaria Ag P. falciparum (HRP2) Card Test	Premier Medical Corporation	32	4,697,300	$0.510	95.0	NA
SD Bioline Malaria Ag P.f/P.v	Standard Diagnostics	18	13,370,905	$0.539	92.0	94.3
SD BIOLINE Malaria Ag P.f	Standard Diagnostics	12	17,177,075	$0.225	95.0	NA
SD BIOLINE Malaria Ag P.f/Pan	Standard Diagnostics	9	1,225,525	$0.668	94.0	91.4
CareStart™ Malaria Pf/Pv (HRP2/pLDH) Ag Combo RDT	Access Bio	4	1,337,805	$0.240	90.8	94.1
CareStart™ Malaria Pf (HRP2) Ag RDT	Access Bio	3	2,715,250	$0.235	91.0	NA
CareStart™ Malaria Pf/PAN (HRP2/pLDH) Ag Combo RDT	Access Bio	3	270,250	$0.486	90.0	94.3
CareStart™ Malaria Pf (HRP2) Ag RDT	Access Bio	2	1,366,550	$0.199	96.0	NA
CareStart™ Malaria Pf (HRP2/pLDH) Ag RDT	Access Bio	2	41,315,625	$0.235	91.0	NA
CareStart™ Malaria Pf/Pv (HRP2/pLDH) Ag Combo RDT	Access Bio	1	532,125	$0.469	91.0	97.1
CareStart™ Malaria Screen RDT	Access Bio	1	525,050	$0.459	93.0	94.3

## Discussion

In summary, the results of the three aims were that (1) lower-quality (< 85% PDS) RDTs have seen decreased market share over this same time period, as have the highest-quality (> 95% PDS) RDTs, though quality of RDTs purchased in 2018 remains high (median 90 to 94.9% PDS) and well above the the WHO minimum standard of 75%; (2) among all four of the linear regression models examining price and quality, there is either no significant relationship (p > 0.05) or a very minimal one (estimate 0.01, p < 0.001) between price per test and panel detection score among RDT purchases in the Global Fund Price and Quality Reporting database from 2009 to 2018, for either *P. falciparum*-detecting RDTs or *P. vivax*-detecting RDTs (respectively). Third, the HHI score for the market overall was 3,570 and was above 2,500 every year of analysis. This would categorize the market as well above the threshold for a “highly concentrated” market by US DOJ standards.

Previous studies have identified related trends that are supported by these results. Similar to Cunningham et al., the results of this analysis demonstrate that quality of RDTs, as defined by PDS, has improved over the course of the WHO-FIND product testing programme [24]. However, RDTs with the highest PDS have become less prevalent over time. So, while the testing programme has encouraged an overall improvement of test quality in the market [24], there has also been a consolidation toward very good—but not the best—RDTs, with some of the highest-quality products leaving the market. There could be several potential reasons for this trend. One is that it is possible quality differences between “very good” RDTs and “the best” RDTs are not significant enough to factor into procurement decisions, even if prices of both groups of RDTs are the same. Previous analyses of malaria RDT procurement indicate that national malaria programmes often purchase several brands of RDTs for use during the same time period, at times up to six different RDTs are being used in the same country [24, 25]. This could indicate that hesitancy regarding brand switching or costly retraining of health care workers to use various RDT brands is in fact not a major driver of buying behavior. Further research is needed to better understand which factors are the most important to purchasers when making decisions about which malaria RDTs to order besides a low price, if quality is not central. This may have implications for producers of RDTs as they consider future product innovation and pricing of RDTs with higher panel detection scores.

One factor that can be ruled out as a reason that the highest-quality RDTs are not purchased is high price. Aim two of this analysis indicates that there is either no or a very minimal relationship between purchase price and quality. If most products are of similar quality, as shown by the RDT market share by quality (Fig. 2), and there is minimal variation in quality for a corresponding increase in price, then this would create very little market space for companies to differentiate their products on quality. This situation would cause companies to compete on price and benefit the lowest-cost producers, likely the largest with economies of scale. Additionally, this could have implications on new product innovation, as manufacturers may see little rationale for further innovation unless the market signals a change in standards or otherwise rewards the innovation.

These results support a well-documented characteristic of the malaria RDT market—it is highly concentrated among a small number of large manufacturers. While economies of scale do provide the advantage of lower prices for consumers, which helps advance the public health goals of improved accessibility of affordable RDTs, long-term sustainability considerations must also be weighed to ensure long-term availability of these products. Economists and regulators would generally prefer to avoid such high degrees of market concentration because they can result in decreased innovation and higher prices due to the lack of competition. In addition, procurement agencies and malaria programmes may have concerns around concentrated markets that result in fewer choices and increased supply chain risks. Several reports by PATH, Unitaid, and the Clinton Health Access Initiative, as well as peer-reviewed publications, have noted the concern around lack of sustainability in the malaria RDT market, for the reasons listed above and others [9, 10, 13, 26]. For example, the purchase price is approaching the cost of manufacturing the RDTs, which creates a market that is not attractive for additional investment and has a high barrier to entry for new manufacturers. If national malaria control programmes are to rely on large private-sector manufacturers to produce products at a near-zero profit margin in the long run, these market conditions put RDT quality and innovation at risk [26]. This was demonstrated in January 2020 when quality control issues with one of the most prominent manufacturers, Access Bio, caused WHO to issue a Notice of Concern warning users about nonconformance with WHO standards and to not place any new orders for products from this company until issues are resolved [39]. Additionally, in 2020 the COVID-19 pandemic disrupted the diagnostics market broadly as the companies with RDT capabilities pivoted resources, which has caused concern about future supply crises. In order to encourage a healthy pipeline of products and create space for new high-quality products, the reasons for this high degree of concentration in the malaria RDT manufacturer space need to be understood.

## Strengths and limitations

There are several important limitations to this analysis. The data in this analysis only included purchase price data reported in the PQR database. This excludes some important purchasing channels such as large single-source contracts and private-sector purchasing. These results may not be generalizable to these other purchasing mechanisms and contexts. The study by Incardona et al. [25 ] found that two-thirds of the responding country representatives described more than one organization being involved with RDT procurement. Thus, these data are incomplete in that they do not describe all RDT purchase orders by national malaria control programmes. However, these data are consistent with reports that summarize other procurement mechanisms such as the VPP and manufacturer-provided estimates [13, 26]. They also represent the largest proportion of transaction information that is publicly available and span nearly a decade of procurement transactions across countries in every WHO region.

There are limitations in the PQR dataset as well. There are often reporting delays in entering of purchase data to the system, and much of the 2018 data may not be included in this analysis. While the Global Fund requires that data be entered into the PQR database in order to receive funding disbursements, the completeness and accuracy of these data are not independently verified. The guidance documents for the PQR database note that in some instances, the reported purchase price of an RDT order includes some supply chain fees, which may inflate the mean price per test [15]. However, since our data examine high-level trends comparing change in average purchase price over time, it is unlikely that these data limitations substantially alter these conclusions.

Despite these limitations, this study still fills important gaps in the literature. This analysis is the first to combine the two largest publicly available datasets of purchase price and product quality data for malaria RDTs. These results describe important trends and relationships within the global market of malaria RDTs that have not yet been characterized and which prompt areas for future study.

## Conclusion

High-quality rapid diagnostic tests are an essential element of any malaria control strategy. Important advancements have been made over the past decade in reducing price, increasing quality, and expanding access to RDTs globally; however, this progress risks stagnation if the health of the RDT market as a whole is neglected. The results of this study provide evidence suggesting that (1) while low-quality malaria RDTs have exited the market, so too have the highest-quality RDTs, (2) there is no or very little relationship between product price and product quality, and (3) the market of malaria RDT manufacturers is highly concentrated around only a few primary suppliers.

Additional research is needed to understand the degree to which price and quality are weighted in decision-making by procurement officers, implications for manufacturers regarding development and pricing of ultra-sensitive RDTs, and if this relationship applies in other malaria RDT procurement channels. This additional research could also lead to identification of similar trends in other vital global health commodities.

### Supplementary Information

**Additional file 1.** Data from the Global Fund including price, quality, volume, RDT product, and country.

**Additional file 2.** Data from eight rounds of WHO-FIND product testing programme, organized by evaluation round.

**Additional file 3.** Data from eight rounds of the WHO-FIND product testing programme, organized by RDT product name, including PDS, heat stability, and false positive rate for each RDT type by evaluation round.

**Additional file 4.** List of countries and corresponding WHO regions.

**Additional file 5.** R script containing code for data cleaning and analyses contained in this manuscript.

## Data Availability

All data generated or analyses during this study are included in this published article as additional files.

## Appendices

### Appendix A. Matching RDT products between WHO-FIND and Global Fund catalogue numbers

**Table:**

Product name (WHO-FIND evaluations)	Catalogue number (global fund)	Rounds evaluated
BIONOTE MALARIA P.f. Ag Rapid Test Kit	RG19-11	3,6
CareStart™ Malaria PAN (pLDH) Ag RDT	RMNM-02591	1,5,8
CareStart™ Malaria Pf (HRP2) Ag RDT	RMOM-03091	7
CareStart™ Malaria Pf (HRP2) Ag RDT j	RMOM-02571	1,5,8
CareStart™ Malaria Pf (HRP2/pLDH) Ag RDT j	RMPM-02571	2,6,8
CareStart™ Malaria Pf/PAN (HRP2/pLDH) Ag Combo RDT	RMRM-02591	8
CareStart™ Malaria Pf/PAN (HRP2/pLDH) Ag Combo RDT j	RMRM-02571	1,5,8
CareStart™ Malaria Pf/PAN (pLDH) Ag RDT j	RMLM-02571	3,7,8
CareStart™ Malaria Pf/Pv (HRP2/pLDH) Ag Combo RDT	RMVM-03091	7
CareStart™ Malaria Pf/Pv (HRP2/pLDH) Ag Combo RDT j	RMVM-02572	2,4,7,8
CareStart™ Malaria Screen RDT	RMAM-05071	3,7
Coretests® One Step Malaria Pf/Pv Ag Test Device	B42-21/B42-22	6
FalciVax™ Rapid Test for Malaria Pv/Pf j	503010025	2,4,6,8
First Response® Malaria Ag P. falciparum (HRP2) Card Test	I13FRC25	1,5
First Response® Malaria Ag. pLDH/HRP2 Combo Card Test	I16FRC	1,2,5
Humasis Malaria P.f/P.v Antigen Test	ANMIV-7025	6
ICT MALARIA DUAL TEST	ML03	3,5,7
ICT MALARIA P.F. CASSETTE TEST	ML01	1,3,7
One Step Malaria HRP2 (P.f) Test	W37-C	2,3,4,6,7
OnSite Malaria Pf Ag Rapid Test	R0114C	2,3,6
OnSite Malaria Pf/Pv Ag Rapid Test	R0112C	2,3,4,6
OptiMAL-IT	710024	1,3
Paracheck Pf rapid test for *P. falciparum* Malaria (Dipstick)	30302025	1,3,4
Paracheck Pf® Rapid Test for Pf Malaria (Ver. 3) j	302030025	1,3,4,8
Parahit f® Ver 1.0—Dipstick	55IC103-50	3,7
Parahit® f Ver 1.0—Device	55IC104-50	3,7
SD BIOLINE Malaria Ag P.f (HRP2/pLDH) j, k	05FK90	3,6,8
SD BIOLINE Malaria Ag P.f/P.f/P.v j, k	05FK120	6,8
SD Bioline Malaria Ag P.f/P.v	05FK80	2,6
SD BIOLINE Malaria Ag P.f/Pan	05FK60	1,3,5
SD BIOLINE Malaria Ag Pf	05FK50	1,5

### Appendix B. Outlier identification process

After joining the PQR and WHO-FIND data, there were outliers in both price per test and PDS in the resulting dataset. There were two small procurement orders (800 and 2,100 RDTs) with prices per test over nine times the size of interquartile range above the mean, which were considered to be outliers and removed from the analysis. Outliers in the PDS scores were also identified and removed. At the beginning of the WHO-FIND product evaluation programme, the WHO recommended minimum quality standard for PDS was > 50% (and in 2012 was raised to 75%) [20]. There were three products procured (across a total of 55 orders) with PDS lower than 50% (for either *P. falciparum* or P. vivax): (1) a *P. falciparum*/Pan test with P. vivax PDS < 50%, (2) a *P. falciparum* /Pan test with *P. falciparum* PDS < 50%, and 3) a *P. falciparum*/P. vivax test with a *P. falciparum* PDS < 50%. For each of these products, data for the low-PDS test line was excluded if that parasite species was not endemic in the purchasing country. For example, Nigeria placed four orders for a two-line *P. falciparum* /*P. vivax* test of which the PDS *P. vivax* score was < 50%; however, *P. vivax* is not considered to be endemic in Nigeria, so for this analysis it was assumed that these tests were procured primarily for the purposes of diagnosing *P. falciparum* infection and excluded the *P. vivax* panel data. Procurement orders that were missing values in the data fields required to calculate price per test (n = 75) were also excluded.

### Appendix C. Market concentration by manufacturer

The table below includes additional details on market share of the top three malaria RDT manufacturers included in this analysis.

**Table:**

Year	HHI score^1^	Market share of top 3 manufacturers
All years	3570	Standard Diagnostics (44.58%) Access Bio (34.38%) Premier Medical Corporation (20.00%) *Other manufacturers (n* = *7, 1.04%)*
2009	3288	Standard Diagnostics (41.45%) Access Bio (28.27%) Premier Medical Corporation (27.69%) *Others (n* = *2, 2.59%)*
2010	4752	Premier Medical Corporation (58.19%) Standard Diagnostics (36.61%) Access Bio (5.06%) *Others (n* = *3, 0.14%)*
2011	3447	Access Bio (41.58%) Standard Diagnostics (32.05%) Premier Medical Corporation (26.28%) *Others (n* = *1, 0.09%)*
2012	3182	Standard Diagnostics (33.31%) Access Bio (32.92%) Premier Medical Corporation (31.41%) *Others (n* = *4, 2.36%)*
2013	3516	Standard Diagnostics (44.91%) Access Bio (32.90%) Premier Medical Corporation (20.34%) *Others (n* = *3, 1.85%)*
2014	3600	Access Bio (41.91%) Standard Diagnostics (39.71%) Premier Medical Corporation (16.29%) *Others (n* = *2, 2.09%)*
2015	4222	Standard Diagnostics (52.56%) Access Bio (36.66%) Premier Medical Corporation (10.77%) *Others (n* = *1, 0.01%)*
2016	7443	Standard Diagnostics (85.27%) Access Bio (13.01%) Premier Medical Corporation (1.72%) *Others (n* = *0)*
2017	4716	Access Bio (57.12%) Standard Diagnostics (37.77%) Premier Medical Corporation (5.10%) *Others (n* = *0)*
2018	3430	Access Bio (39.84%) Premier Medical Corporation (34.15%) Standard Diagnostics (26.00%) *Others (n* = *0)*

^1^ The US DOJ considers an HHI score below 1,500 as an unconcentrated market, between 1,500 and 2,500 as a moderately concentrated market, and above 2,500 as a highly concentrated market

### Appendix D. Complete datasets and files

Additional files available

**Table:**

File Title	File Name	File Type	Description
Global Fund PQR Data	“globalfund_df.csv”	CSV	Data from the Global Fund including price, quality, volume, RDT product, and country (Additional file 1)
Adjusted PDS Data	“pds_adj_df.csv”	CSV	Data from eight rounds of WHO-FIND product testing programme, organized by evaluation round (Additional file 2)
WHO-FIND Data	“find_df.csv”	CSV	Data from eight rounds of the WHO-FIND product testing programme, organized by RDT product name, including PDS, heat stability, and false positive rate for each RDT type by evaluation round (Additional file 3)
WHO Regions	“who_region_df.csv”	CSV	List of countries and corresponding WHO regions (Additional file 4)
Analysis Script	“Malaria RDT Analysis Script.R”	R	R script containing code for data cleaning and analyses contained in this manuscript (Additional file 5)

## Abbreviations

FIND: Foundation for Innovative New Diagnostics
Global Fund: The Global Fund to Fight AIDS, Tuberculosis and Malaria
HHI: Herfindahl–Hirschman Index
HIV: Human immunodeficiency virus
PDS: Panel detection score
PPM: Pooled Procurement Mechanism
PQR: Global Fund’s Price and Quality Reporting
RDT: Rapid diagnostic test
USD: US dollar
US DOJ: US Department of Justice
WHO: World Health Organization
